# Effects of Homofermentative and Heterofermentative *Lactobacillus* on Fermentation and Bacterial Communities of Sweet Corn By-Product Silage

**DOI:** 10.3390/ani16182970

**Published:** 2026-09-21

**Authors:** Youyan Tao, Fuhou Li, Yanwei Zhang, Hongyu Tang, Yuchong Wang, Ning Ma, Meng Yu, Peng Wang

**Affiliations:** 1 College of Animal Sciences, Jilin University, Changchun 130062, China; yytao24@mails.jlu.edu.cn (Y.T.); lifh@jlu.edu.cn (F.L.); yanwei24@mails.jlu.edu.cn (Y.Z.); tanghy@jlu.edu.cn (H.T.); yumeng22@mails.jlu.edu.cn (M.Y.); 2 Siping Academy of Modern Agricultural Sciences, Siping 136500, China; wangyuc212026@163.com; 3 Dehui Urban District Animal Husbandry and Veterinary Workstation, Changchun 130300, China; shyi5678@126.com

**Keywords:** fermentation quality, lactic acid bacteria additive, bacterial community, sweet corn by-product silage

## Abstract

To investigate the effects of different lactic acid bacteria additives on the bacterial community structure of silage prepared from fresh corn processing by-products, three treatment groups, including a non-inoculated control group (C), a *Lactiplantibacillus plantarum*-supplemented group (P), and a *Lentilactobacillus buchneri*-supplemented group (B), were set up in this study. Fermentation quality and bacterial community dynamics were measured on days 1, 3, 7, 15, and 45 of ensiling, while nutritional components, *in vitro* digestibility, and aerobic stability of the silage were systematically analyzed at the end of the 45-day ensiling period. The results showed that pH and ammonia nitrogen (NH_3_-N) contents in all groups gradually decreased with the prolongation of ensiling duration. Lactic acid (LA) and acetic acid (AA) contents increased progressively over the ensiling period, and their responses were significantly influenced by additives × day interactions (*p* < 0.05), with group P showing higher levels than group B on days 15 and 45. Propionic acid (PA) and butyric acid (BA) were not detected in any group. After 45 days of ensiling, the organic matter (OM) content in group B was significantly higher than that in the other two groups (*p* < 0.05). Compared with group C, the acid detergent lignin (ADL) content was significantly lower in both groups P and B (*p* < 0.05). Group P presented significantly higher *in vitro* organic matter digestibility (IVOMD), *in vitro* crude protein digestibility (IVCPD), and *in vitro* neutral detergent fiber digestibility (IVNDFD) than groups C and B (*p* < 0.05). In contrast, group B showed greater *in vitro* dry matter digestibility (IVDMD) than group P (*p* < 0.05). All experimental groups maintained silage aerobic stability exceeding 72 h, with no significant differences detected among treatments. The bacterial community of sweet corn by-product silage changed substantially after ensiling. Bacterial community diversity gradually decreased as ensiling proceeded. At 45 days of ensiling, the dominant bacterial genera shifted from *Weissella*, *Levilactobacillus*, and *Pediococcus* to *Levilactobacillus* and *Lentilactobacillus*. On days 1, 7, and 15 of ensiling, differences in the relative abundances of specific bacterial taxa were observed among the three groups. At specific ensiling time points, inoculation with *L. plantarum* was associated with higher relative abundances of most dominant bacterial taxa in sweet corn by-product silage than inoculation with *L. buchneri*.

## Simple Summary

This study explored the effects of homofermentative and heterofermentative lactic acid bacteria on fermentation quality, chemical composition, *in vitro* digestibility, and succession of bacterial communities during ensiling of fresh sweet corn processing by-products. The results indicate that *Lactiplantibacillus plantarum* inoculation was associated with greater changes in fermentation characteristics and bacterial community composition compared with *Lentilactobacillus buchneri*. These findings offer a preliminary reference for the potential utilization of sweet corn processing by-products as alternative feed resources.

## 1. Introduction

Fresh sweet corn (*Zea mays* L. var. *saccharata*) refers to corn harvested at the milk stage for direct consumption and is characterized by high soluble sugar content [1]. According to the *2022 China White Paper on Fresh Sweet Corn Consumption Trends*, China’s fresh sweet corn production has grown rapidly in recent years, with a compound annual growth rate of 13.46% [2]. As market demand expands and processing methods diversify, large amounts of by-products, including husks, cobs, corn silk, and damaged kernels, are generated, constituting 20–30% of the total raw material yield [3]. These by-products are rich in starch, soluble sugars, and cellulose [4,5], yet their high moisture content complicates preservation. Direct ensiling of such materials may increase the risk of undesirable *Clostridium* fermentation and spoilage. Since silage fermentation quality is affected by multiple factors, including fermentable substrate concentration, buffering capacity, epiphytic microbial communities, and ensiling conditions [6], a more systematic evaluation of this feed resource is required.

Under high-moisture conditions, spoilage microorganisms such as yeasts, molds, and *Clostridium* multiply rapidly, causing elevated concentrations of ammonia nitrogen and butyric acid, retarding the pH decline, and ultimately compromising silage quality [7]. To address this challenge, mixing forage with low-moisture by-products (e.g., wheat bran or rice husks) prior to ensiling is a commonly adopted strategy to reduce moisture content, thereby suppressing mold proliferation and enhancing fermentation stability [8]. In addition, exogenous microbial inoculants are often used to modulate the microbial community during fermentation, regulating the ensiling process and preserving the forage. Specifically, lactic acid bacteria with distinct metabolic types exert divergent functions in fermentation systems. *Lactiplantibacillus plantarum* (*L. plantarum*), a representative homofermentative strain commonly used in silage production, ferments rapidly to produce lactic acid, thereby quickly lowering pH, effectively inhibiting the growth of undesirable microorganisms, and reducing dry matter losses [9,10]. In contrast, heterofermentative lactic acid bacteria consume more substrates to achieve equivalent acidification and exhibit slightly lower acid production efficiency, but they can produce antimicrobial substances such as acetic acid to enhance aerobic stability [11]. For example, *Lentilactobacillus buchneri* (*L. buchneri*) is a typical heterofermentative strain that enhances resistance to aerobic spoilage through the production of acetic acid and 1,2-propanediol [12]. Furthermore, silage fermentation essentially involves dynamic microbial community succession. Therefore, although organic acid profiles and other physicochemical properties are commonly used to evaluate inoculation effects, measuring only these end-products is insufficient to adequately characterize the microbial processes underlying these effects. Previous studies have demonstrated that inoculation with strains differing in fermentation type (homofermentative or heterofermentative) can influence bacterial community succession during the ensiling of whole-plant maize [13], oats [14], alfalfa [15], and other forages. However, studies investigating the effects of different lactic acid bacteria on the ensiling of fresh sweet corn processing by-products—an unconventional feed resource—remain scarce. Although multi-strain lactic acid bacterial inoculants are widely used in commercial silage production, understanding the fermentation behavior of individual strains in specific substrates is key to deciphering inter-strain interactions. We hypothesize that the two strains, driven by their contrasting fermentation pathways, may lead to distinctly different fermentation processes and bacterial community succession: *L. plantarum* accelerating early acidification, and *L. buchneri* producing acetic acid in the later stage to inhibit spoilage microorganisms and enhance aerobic stability. Such differences are expected to translate into measurable differences in organic acid profiles, aerobic stability, nutrient preservation, and bacterial community dynamics.

Based on the above considerations, this study investigated fresh sweet corn processing by-products mixed with wheat bran and inoculated with homofermentative (*L. plantarum*) or heterofermentative (*L. buchneri*) lactic acid bacteria to systematically evaluate their effects on fermentation quality, chemical composition, *in vitro* digestibility, and aerobic stability. In parallel, the effects of inoculant type and ensiling duration on microbial community dynamics during ensiling were characterized via high-throughput sequencing.

## 2. Materials and Methods

### 2.1. Raw Materials

The lactic acid bacteria strains used in this study were *L. plantarum* LP1 and *L. buchneri* 9-2. *L. plantarum* LP1 was obtained from Sapporo Snow Brand Seed Co., Ltd. (Sapporo, Japan), while *L. buchneri* 9-2 was kindly provided by the Probiotics and Biofeed Research Center, Lanzhou University, China. Both strains were provided in lyophilized powder form. Fresh sweet corn processing by-products and wheat bran were collected from Jilin Xinyu Biotechnology Co., Ltd. (Jilin, China) These by-products were derived exclusively from sweet corn grown for fresh consumption. Their main components were corn husks and corn cobs, with corn silk and broken kernels collectively accounting for less than 3% of the total weight. These materials represented typical mixed by-products of fresh sweet corn processing, with a crushed particle size of 0.5–1.5 cm. The chemical composition, buffering capacity (BC), and gross energy (GE) of fresh sweet corn by-products and wheat bran before ensiling are summarized in Table 1. All determinations were conducted in quadruplicate.

### 2.2. Experimental Design

Based on preliminary trials, crushed fresh sweet corn by-products were mixed with wheat bran at a ratio of 8:2 (fresh basis) to achieve optimal fermentation performance [16]. The resulting mixture had a moisture content of approximately 65%, calculated from the initial moisture contents of the two raw materials. The experiment consisted of three treatments: (1) control (C), no lactic acid bacteria inoculation; (2) P, inoculated with *L. plantarum* LP1 at 5 × 10^6^ CFU/g fresh matter (FM); and (3) B, inoculated with *L. buchneri* 9-2 at the same inoculation dose. The lyophilized strains were resuspended in distilled water following the manufacturer’s instructions and uniformly sprayed onto the forage samples using a mini spray bottle. The samples were then thoroughly mixed manually to ensure uniform inoculum distribution. The total water application volume was maintained at 20 mL/kg FM, and the control group received an equivalent volume of sterile distilled water to account for the effect of water addition. Approximately 500 g of the well-mixed raw material was sealed in polyethylene silage bags (250 mm × 350 mm) using a vacuum sealer and stored at ambient temperature (20.5 ± 1.5 °C). The experiment was arranged in a 3 × 5 factorial design, comprising three inoculation treatments and five ensiling durations (1, 3, 7, 15, and 45 days). Each treatment had four biological replicates at each sampling time point, yielding a total of 60 independent silage bags. A destructive sampling method was adopted in this experiment, whereby individual silage bags were opened at each sampling time point. For the aerobic stability evaluation, an additional six bags per treatment (18 bags in total) were prepared and ensiled for 45 days.

### 2.3. Analysis

#### 2.3.1. Fermentation Quality Analysis

A 20 g subsample of silage was combined with 180 mL of distilled water, fully homogenized, and stored at 4 °C overnight for aqueous extraction. The mixture was then filtered through quantitative filter paper [17]. The pH of the filtrate was measured using a digital pH meter (PHSJ-4F, Yidian Group Co., Ltd., Shanghai, China). Ammonia nitrogen (NH_3_-N) concentration was determined by steam distillation, and the NH_3_-N to total nitrogen (TN) ratio was subsequently calculated [18]. Organic acids were analyzed according to the method described by Cao et al. [19]. Lactic acid (LA), acetic acid (AA), propionic acid (PA), and butyric acid (BA) were quantified using high-performance liquid chromatography (HPLC) equipped with a Shodex RS Pak KC-811 column (Showa Denko KK, Kawasaki, Japan) and a diode array detector (SPD-20A, Shimadzu Corporation, Kyoto, Japan). The mobile phase was 3 mmol/L perchloric acid, with a flow rate of 1.0 mL/min, column temperature of 50 °C, injection volume of 10 µL, and detection wavelength of 210 nm.

#### 2.3.2. Chemical Composition Analysis

Silage samples and fresh raw materials were oven-dried to a constant weight in a forced-air oven (DHG-9140A, Shanghai Yiheng Scientific Instrument Co., Ltd., Shanghai, China) at 60 °C for 72 h. The dried samples were ground with a pulverizer (WK-150A, Qingzhou Maidesen Pharmaceutical Machinery Factory, Weifang, China), sieved through a 1 mm screen, and stored in sealed plastic bags.

Dry matter (DM, AOAC 934.01), organic matter (OM, AOAC 942.05), and crude protein (CP, AOAC 990.03) were determined according to the official methods of the Association of Official Analytical Chemists (AOAC), while gross energy (GE) was measured using an oxygen bomb calorimeter [20]. Neutral detergent fiber (NDF), acid detergent fiber (ADF), and acid detergent lignin (ADL) contents were quantified using a fiber analyzer (YT-SF20, LanHong Optoelectronics Technology Co., Ltd., Weifang, China) following the method of Van Soest et al. [21]. No heat-stable α-amylase or sodium sulfite was applied during NDF determination. Water-soluble carbohydrate (WSC) content was determined via the anthrone–sulfuric acid colorimetric method [22]. Buffering capacity (BC) was measured following the procedure reported by Playne and McDonald [23].

#### 2.3.3. *In Vitro* Digestibility Analysis

All animal-related procedures in this study were reviewed and approved by the Animal Ethics Committee of Jilin University (approval number: SY20209600; approval date: 2 January 2024). A subsample of approximately 0.5 g of oven-dried silage was weighed into a filter bag (ANKOM F57, ANKOM Technologies, Macedon, NY, USA), heat-sealed using a bag sealer, and then transferred into a 130-mL screw-cap tube for *in vitro* digestion. The *in vitro* digestion procedure was performed following Goeser et al. [24], with minor modifications. Rumen fluid was collected from four 10-month-old F1 crossbred sheep (German Meat Merino × Small-tailed Han) fitted with permanent rumen cannulas. The sheep were fed a corn silage-based diet twice daily (dietary formulation and chemical composition are shown in Table A1). Rumen fluid was collected 1 h before morning feeding, pooled in equal volumes from the four donors, filtered through four layers of cheesecloth, transferred to a pre-warmed thermos flask (39 °C), and continuously purged with CO_2_. A buffer solution was prepared following the method of Longland et al. [25], and mixed with the pooled rumen fluid at a ratio of 1:4 (buffer: rumen fluid) to prepare the inoculum. Each digestion tube received 50 mL of the inoculum. The digestion tubes containing the inoculum and samples were incubated at 39 °C in a shaking water bath for 48 h. After incubation, the filter bags were removed, rinsed with cold distilled water, dried at 100 °C for 24 h, and weighed. The dried residues were ground and analyzed for CP, GE, NDF, and ash using the same analytical procedures applied to the original silage samples. Each silage bag was defined as the experimental unit for *in vitro* digestibility measurements; one incubation tube was prepared per bag. Each treatment had four biological replicates (*n* = 4), each derived from an individual silage bag. All replicates were assayed once within a single incubation run. After CP, GE, NDF, and ash determination, the *in vitro* DM digestibility (IVDMD), *in vitro* OM digestibility (IVOMD), *in vitro* CP digestibility (IVCPD), *in vitro* GE digestibility (IVGED), and *in vitro* NDF digestibility (IVNDFD) were calculated following the formula:

$$D(\%)=\frac{w_{1}-w_{2}}{w_{1}}\times 100$$

where D is the *in vitro* nutrient digestibility, W_1_ is the nutrient content before *in vitro* digestion, and W_2_ is the nutrient content after *in vitro* digestion.

#### 2.3.4. Aerobic Stability Analysis

Aerobic stability refers to the resistance of silage to aerobic deterioration and is defined as the time required for silage temperature to exceed ambient temperature by 2 °C [26]. After 45 days of ensiling, the aerobic stability of the silage was evaluated. For each treatment, six additional bags were prepared to compensate for potential bag damage. From these, four bags with intact seals and uniform appearance were selected for the aerobic stability test. The contents of each selected bag were transferred into an individual 1-L plastic bucket (i.e., four replicates per treatment, with each bucket corresponding to one independent silage bag). All buckets were covered with four layers of sterile gauze to minimize airborne dust contamination and then stored at an average ambient temperature of 20.5 ± 1.5 °C. A thermocouple probe connected to a multi-channel automatic temperature recorder (HY-R4000, Hongyi Automation Instrument Co., Ltd., Huai’an, China) was inserted vertically into the center of the silage mass in each bucket. Silage temperature was recorded hourly for 7 consecutive days under aerobic conditions. Ambient temperature was recorded hourly using the same temperature recorder throughout the 7-day exposure period.

#### 2.3.5. 16S rRNA Sequencing and Data Analysis

A 10 g silage sample or a 15 g fresh raw material sample was transferred into an Erlenmeyer flask containing 90 mL of sterile saline solution and then shaken on a reciprocating shaker (120 rpm, 25 °C) for 15 min. The suspension was filtered through four layers of sterile gauze and then centrifuged at 12,000 rpm for 10 min to harvest the microbial pellet. Total genomic DNA was extracted with a commercial DNA extraction kit (DP302-02, Tiangen Biochemical Technology Co., Ltd., Beijing, China). DNA purity and concentration were determined using a NanoDrop 2000 spectrophotometer (Thermo Fisher Scientific, Inc., Madison, WI, USA).

The nearly full-length 16S rRNA gene, covering hypervariable regions V1 to V9, was amplified using the universal primers 27F (5′-AGAGTTTGATCMTGGCTCAG-3′) and 1492R (5′-ACCTTGTTACGACTT-3′). Negative controls were included in all amplification procedures. Library construction and PacBio sequencing were performed by Shanghai Personalbio Technology Co., Ltd. (Shanghai, China) on the PacBio Sequel III platform (Pacific Biosciences, Menlo Park, CA, USA). Subsequent bioinformatic analysis was conducted following the method described by Dong et al. [27]. Raw sequencing data were processed via the Personalbio GenesCloud platform. Circular consensus sequencing (CCS) reads were generated from raw subreads using the CCS algorithm (v4.0.0). The reads were demultiplexed according to the barcode sequences of individual samples. The numbers of raw and clean reads, as well as the sequencing depth per sample, are listed in Table A2. For the ASV-based analytical pipeline, primer trimming, quality filtering, denoising, and chimera removal were performed using the DADA2 plugin in QIIME 2 (v2019.4) to obtain amplicon sequence variants (ASVs) and the corresponding abundance table. For the OTU-based pipeline, high-quality sequences were clustered into operational taxonomic units (OTUs) at a 97% similarity threshold using VSEARCH (v2.13.4), and chimeras were eliminated via the UCHIME uchime_denovo module. Singleton ASVs and OTUs were discarded, and both datasets were rarefied to the minimum sequencing depth for standardization. Bacterial community composition, alpha diversity, beta diversity, taxonomic comparisons, and linear discriminant analysis effect size (LEfSe) analysis were conducted using the ASV dataset. Taxonomic assignment was performed using the NCBI RefSeq 16S rRNA database with a minimum confidence cutoff of 0.7. The OTU table was exclusively used as the input file for PICRUSt2 (v2.5.2) functional prediction. Functional potential was predicted using default parameters against the KEGG Orthology (KO) database. The raw 16S rRNA gene sequencing reads were deposited in the NCBI Sequence Read Archive (SRA) under the BioProject accession number PRJNA1522123.

### 2.4. Statistical Analysis

Each silage bag served as the experimental unit for all measured variables. Statistical analyses were performed using SPSS 26.0 (IBM Corp., Armonk, NY, USA), with significance set at *p* < 0.05. For data collected at multiple time points, two-way analysis of variance (ANOVA) was applied, with ensiling day, treatment, and their interaction as fixed effects, followed by Tukey’s HSD test for multiple comparisons. For data collected at a single time point, one-way ANOVA was performed, followed by Tukey’s HSD test. For Shannon index boxplots, the Kruskal–Wallis test followed by Dunn’s post-hoc test was used for multiple comparisons at each time point (*p* < 0.05). Beta diversity was assessed via PERMANOVA based on the Bray–Curtis distance matrix, with treatment, ensiling day, and their interaction as fixed factors, with 9999 permutations implemented using the vegan package (v2.7-6) in R (v4.3.3). Linear discriminant analysis effect size (LEfSe) was performed using the LEfSe package (v1.1.2) in Python (v3.9), incorporating Kruskal–Wallis and Wilcoxon tests, with an LDA score threshold > 2.

## 3. Results

### 3.1. Fermentation Quality

**Figure 1:**
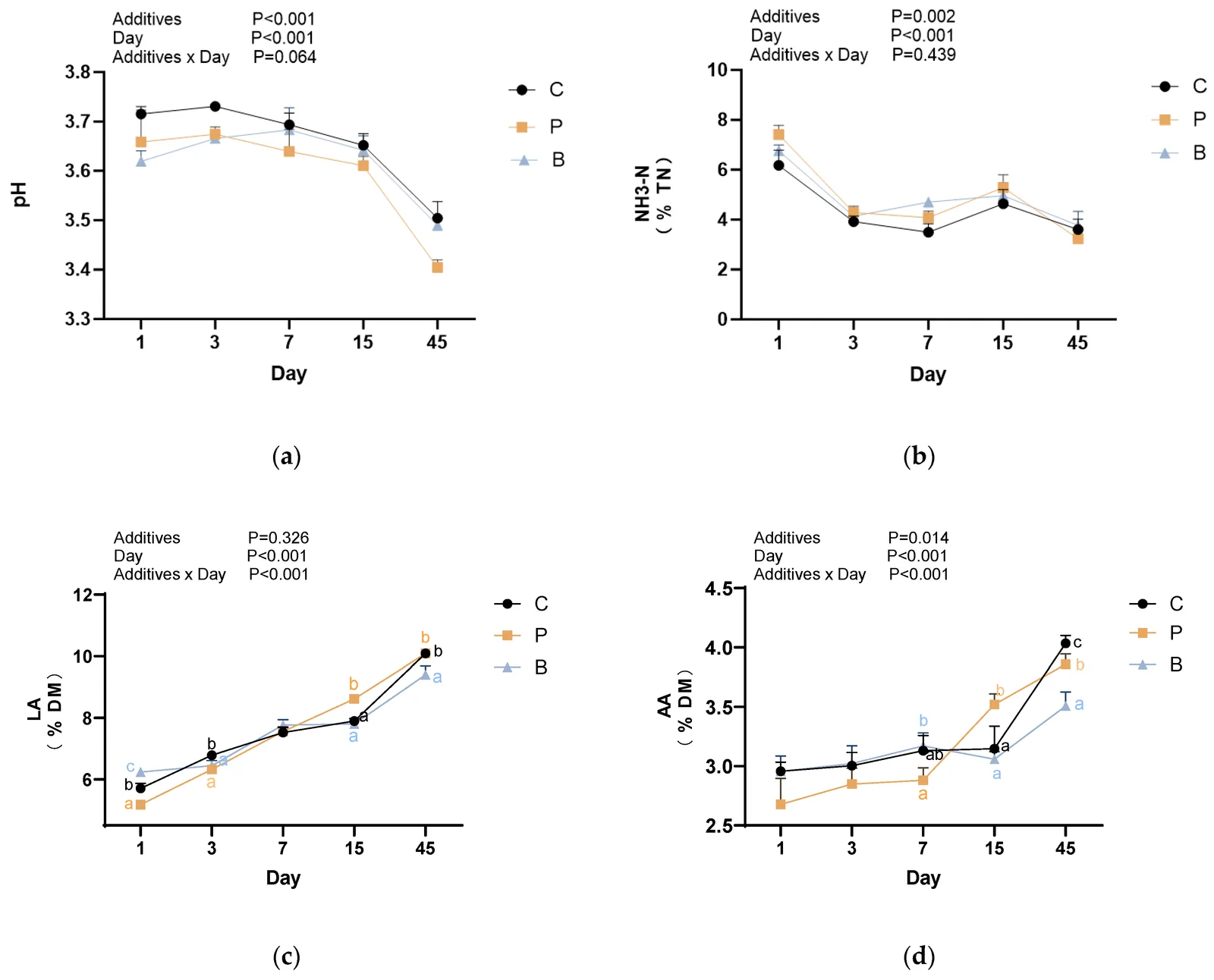
Fermentation characteristics of fresh sweet corn by-product silage. (**a**) pH value; (**b**) NH_3_-N concentration; (**c**) LA concentration; (**d**) AA concentration. C, control, no lactic acid bacteria inoculation; P, inoculated with *L. plantarum* LP1 at 5 × 10^6^ CFU/g fresh matter; B, inoculated with *L. buchneri* 9-2 at 5 × 10^6^ CFU/g fresh matter. Day, ensiling days; Additives, microbial inoculants; and Additives × Day, the interaction between ensiling days and additives. Different lowercase letters at the same ensiling time indicate significant differences among treatments (*p* < 0.05). For pH and NH_3_-N, the Additives × Day interaction was not significant (*p* > 0.05); therefore, only main effects are reported, and no within-day treatment comparisons are shown. Data were analyzed by two-way analysis of variance (ANOVA) followed by Tukey’s HSD test for multiple comparisons.

As shown in Figure 1, the pH of all treatments decreased with ensiling time, and both ensiling day and additive had significant effects on pH (*p* < 0.001), with the P and B groups generally showing lower pH values than the control. NH_3_-N concentration was also significantly affected by ensiling day and additive (*p* < 0.001 and *p* = 0.002, respectively), and all treatment groups reached their lowest values at 45 days of ensiling. The additives × day interactions for pH and NH_3_-N were not significant (*p* = 0.064 and *p* = 0.439, respectively). Lactic acid (LA) and acetic acid (AA) concentrations increased significantly with ensiling time (*p* < 0.001), and significant additives × day interactions were observed for both organic acids (*p* < 0.001), indicating that additive effects varied over time. During the first 15 days of ensiling, LA and AA concentrations in group P were significantly higher than those in groups C and B (*p* < 0.05). At 45 days of ensiling, group P still exhibited higher LA and AA concentrations than group B (*p* < 0.05).

### 3.2. Chemical Composition

No significant differences in DM, CP, NDF, and ADF concentrations were found among treatments (Table 2). However, group B had significantly greater OM concentration than groups C and P (*p* < 0.05). Both inoculant treatments had significantly lower ADL concentrations than group C (*p* < 0.05).

### 3.3. In Vitro Digestibility

The *in vitro* digestibility of silage is presented in Table 3. The IVDMD in group B was significantly higher than that in group P (*p* < 0.05). In contrast, the IVOMD, IVCPD, and IVNDFD in group P were significantly higher than those in groups C and B (*p* < 0.05).

### 3.4. Aerobic Stability

**Figure 2:**
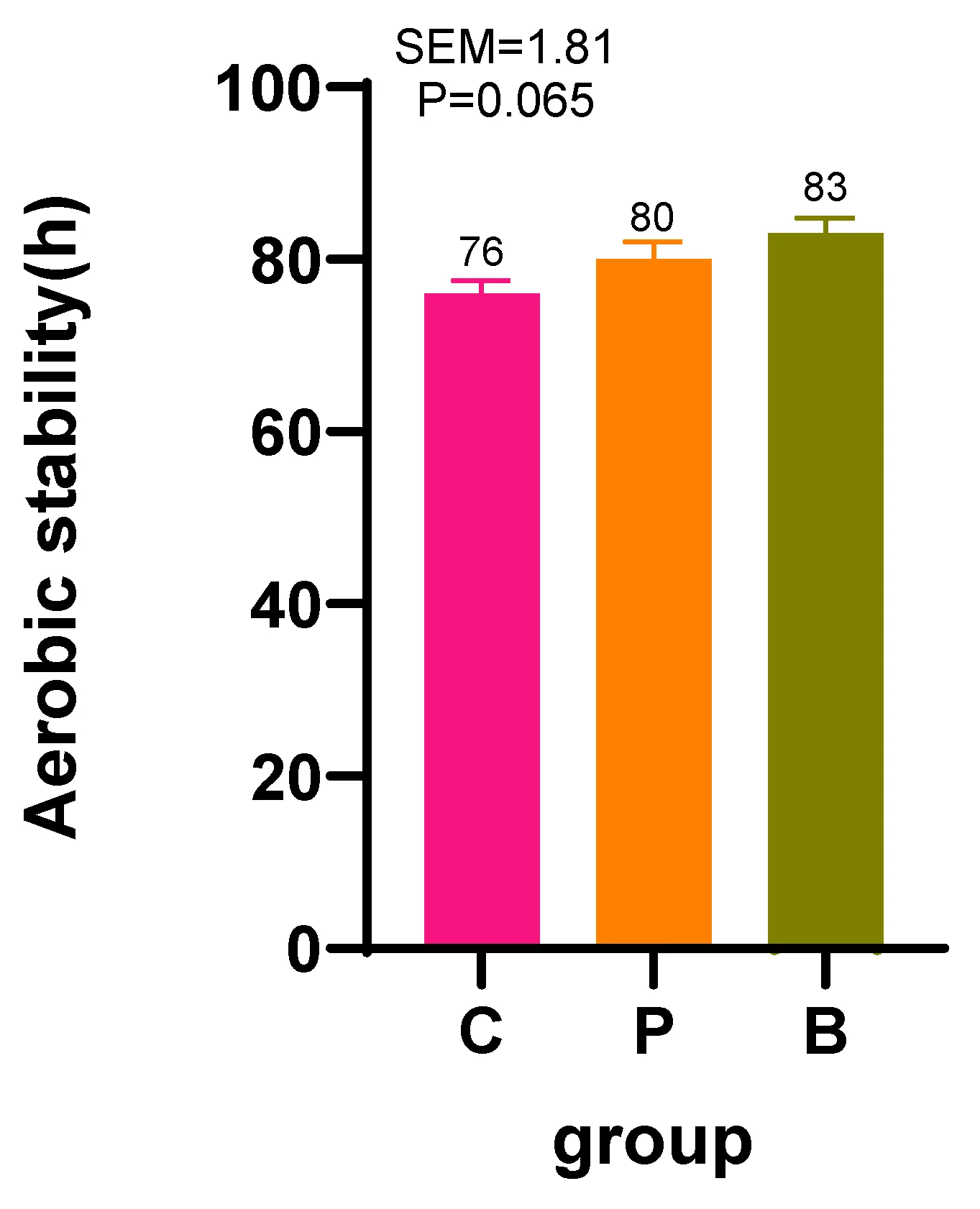
Aerobic stability of silages. C, control, with no lactic acid bacteria inoculation; P, silage inoculated with *L. plantarum* LP1; B, silage inoculated with *L. buchneri* 9-2. Samples are labeled accordingly (e.g., C1, P1, B1 for day 1; C3, P3, B3 for day 3; etc.). Inoculation dose: 5 × 10^6^ CFU/g fresh matter. Data were analyzed by one-way ANOVA followed by Tukey’s HSD test for multiple comparisons.

The time required for the silage temperature to exceed ambient temperature by 2 °C is shown in Figure 2. The aerobic stability duration for C, P, and B was 76, 80, and 83 h, respectively. All treatments exceeded 72 h, with no significant differences among them.

### 3.5. Composition of Bacterial Communities

**Figure 3:**
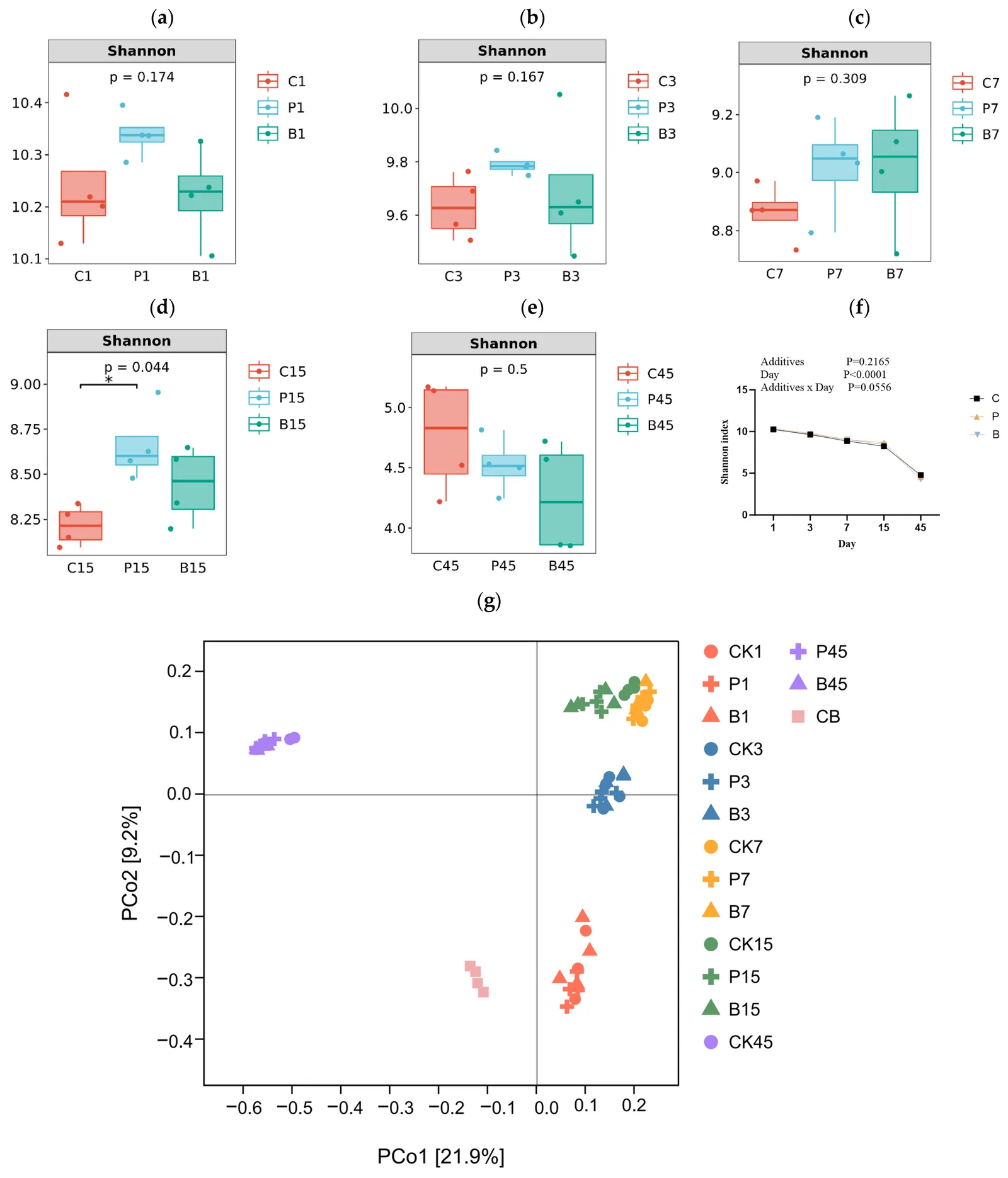
Bacterial α-diversity (Shannon index) during silage fermentation and principal coordinate analysis (PCoA) of bacterial community structure in silage. Boxplots (**a**–**e**) show the distribution of the Shannon index in silage on days 1, 3, 7, 15, and 45, respectively. Figure (**f**) is a line graph depicting the temporal trend of the Shannon index during silage fermentation. Figure (**g**) is a PCoA plot based on Bray–Curtis dissimilarity showing bacterial community structure. The percentage of variation explained by each principal coordinate is indicated in parentheses. C, control, with no lactic acid bacteria inoculation; P, silage inoculated with *L. plantarum* LP1; B, silage inoculated with *L. buchneri* 9-2. Samples are labeled accordingly (e.g., C1, P1, B1 for day 1; C3, P3, B3 for day 3; etc.). Inoculation dose: 5 × 10^6^ CFU/g fresh matter. Data in (**a**–**e**) were analyzed by Kruskal–Wallis + Dunn’s test; data in (**f**) by two-way ANOVA + Tukey’s HSD; data in (**g**) by PERMANOVA based on Bray–Curtis distances (treatment, ensiling day, and their interaction; 9999 permutations). * stands for 0.01 < *p* ≤ 0.05.

As shown in Figure 3f, the α-diversity (Shannon index) of the bacterial communities in all groups decreased significantly with increasing ensiling time (*p* < 0.0001), reaching the lowest value at the end of ensiling.

PCoA was used to visualize the effects of *L. plantarum* and *L. buchneri* on silage bacterial beta diversity. PERMANOVA based on Bray–Curtis distances showed that ensiling day significantly affected bacterial community composition (R^2^ = 0.392, *p* = 0.0001), whereas the effects of treatment (R^2^ = 0.024, *p* = 0.264) and the treatment × day interaction (R^2^ = 0.095, *p* = 0.219) were not significant. The first two principal coordinates accounted for 21.9% and 9.2% of the total variation, respectively. As shown in Figure 3g, samples from different silage durations formed distinct clusters, with fresh and ensiled samples clearly separated from each other.

Taxon-level comparisons of relative abundance were exploratory, and no false discovery rate (FDR) correction was applied across taxa.

**Figure 4:**
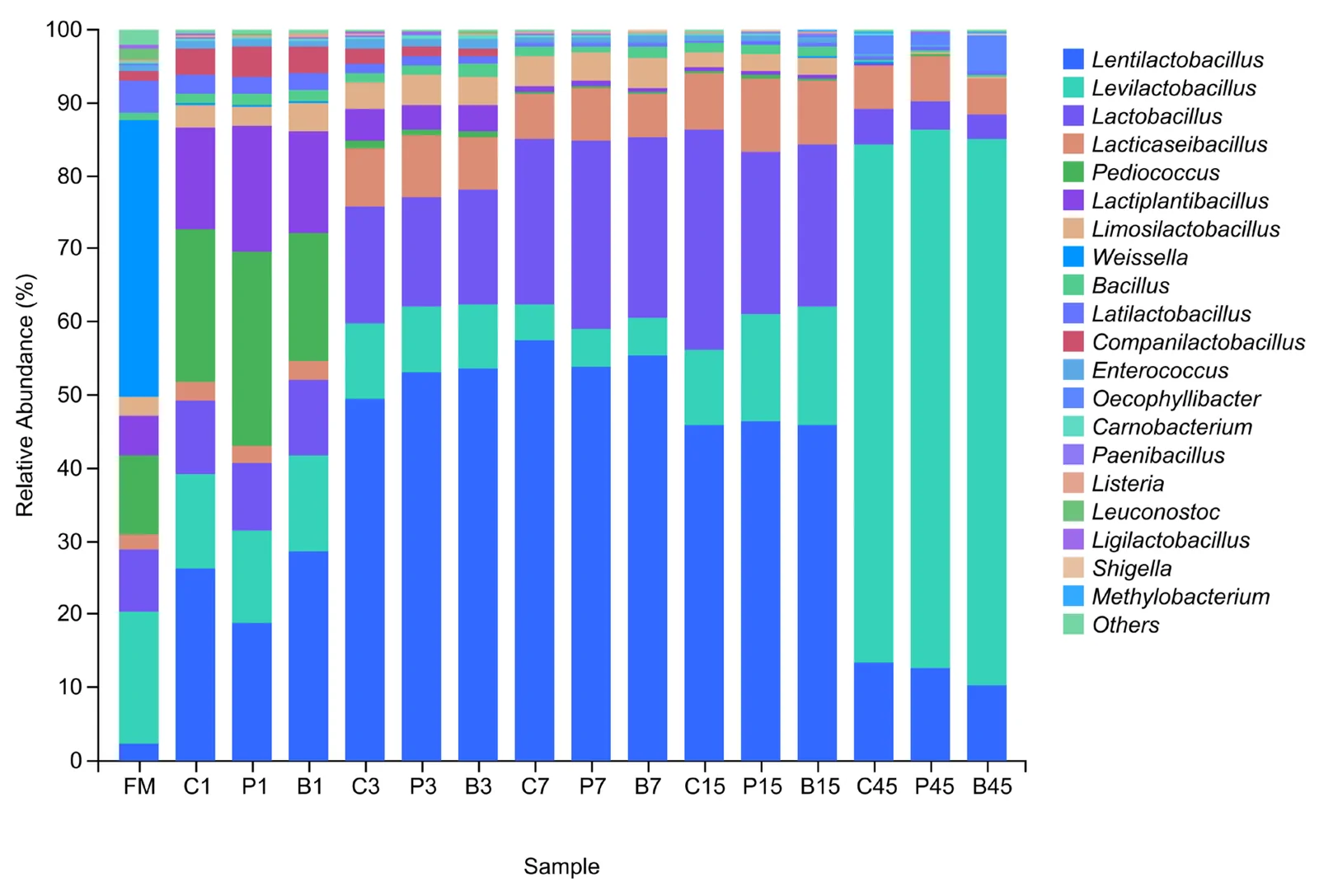
Bacteria at genus-level of silage. FM, fresh matter; C, control, with no lactic acid bacteria inoculation; P, silage inoculated with *L. plantarum* LP1; B, silage inoculated with *L. buchneri* 9-2. Samples are labeled accordingly (e.g., C1, P1, B1 for day 1; C3, P3, B3 for day 3; etc.).

Figure 4 presents the bacterial community composition at the genus level. In fresh materials, *Weissella* (37.82%), *Levilactobacillus* (18.06%), *Lactobacillus* (8.58%), *Pediococcus* (10.86%), and *Lactiplantibacillus* (5.62%) were the predominant genera. Shifts in bacterial community composition were observed after ensiling. The relative abundance of *Weissella* sharply decreased after one day of ensiling, whereas the relative abundances of *Pediococcus* and *Lentilactobacillus* increased rapidly. However, *Pediococcus* was replaced by other genera on day 3, while *Lentilactobacillus* gradually declined during the late ensiling period. After 45 days of fermentation, *Levilactobacillus* became the dominant genus.

**Figure A1:**
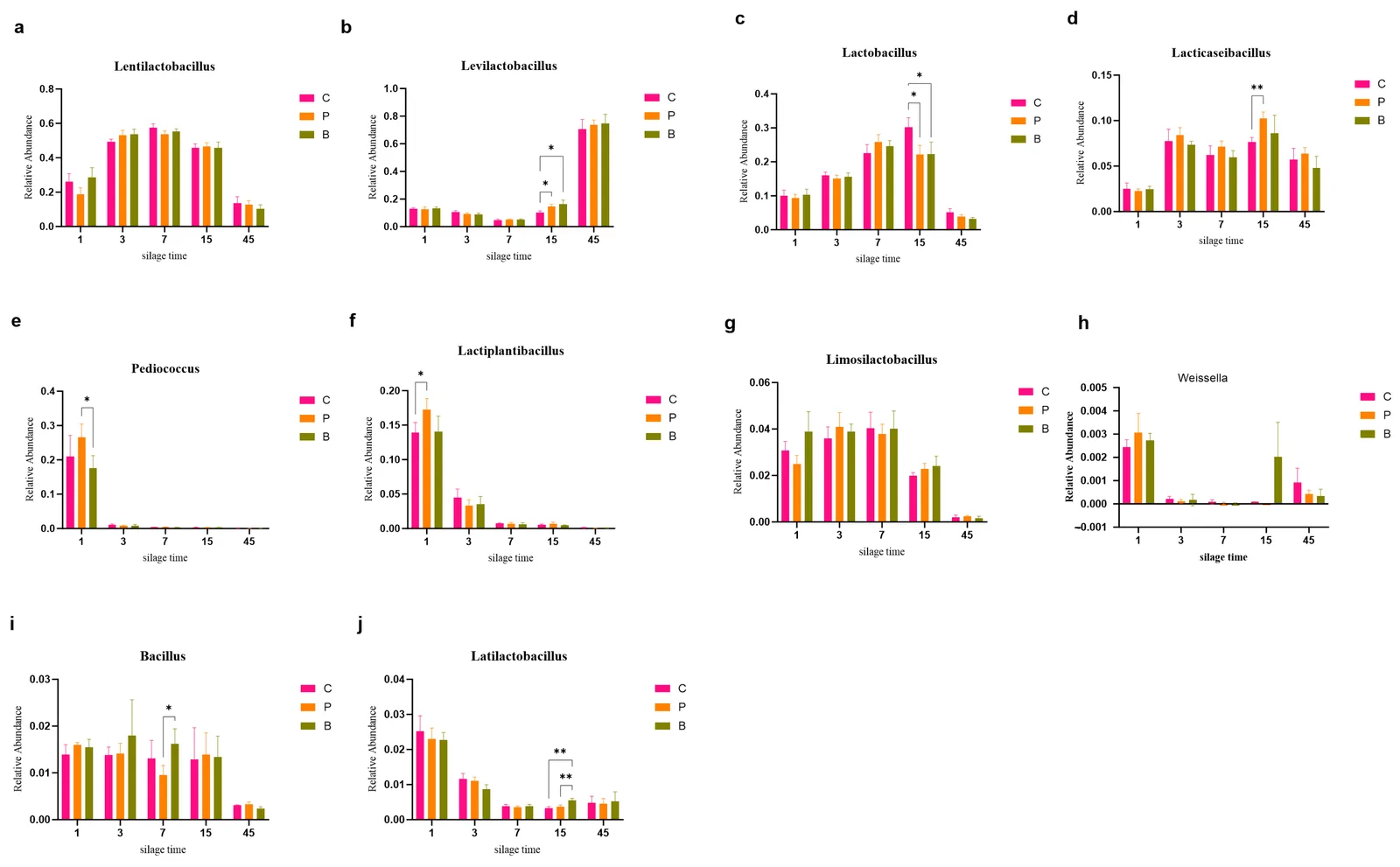
Relative abundance of dominant bacterial genera in silage. Panels (**a**–**j**) show the relative abundance (mean ± SD, n = 4) of ten bacterial genera: (**a**) *Lentilactobacillus*, (**b**) *Levilactobacillus*, (**c**) *Lactobacillus*, (**d**) *Lacticaseibacillus*, (**e**) *Pediococcus*, (**f**) *Lactiplantibacillus*, (**g**) *Limosilactobacillus*, (**h**) *Weissella*, (**i**) *Bacillus*, (**j**) *Latilactobacillus*. C, control, with no lactic acid bacteria inoculation; P, silage inoculated with *L. plantarum* LP1; B, silage inoculated with *L. buchneri* 9-2. Samples are labeled accordingly (e.g., C1, P1, B1 for day 1; C3, P3, B3 for day 3; etc.). Inoculation dose: 5 × 10^6^ CFU/g fresh matter. Data were analyzed by one-way ANOVA followed by Tukey’s HSD test for multiple comparisons. * indicates 0.01 < *p* ≤ 0.05; ** indicates 0.001 < *p* ≤ 0.01.

Figure A1 shows the relative abundances of dominant bacterial genera in silage. On ensiling day 1, group P showed higher relative abundances of *Pediococcus* (vs. group B) and *Lactiplantibacillus* (vs. group C) (unadjusted *p* < 0.05). The relative abundance of *Bacillus* was elevated in group B at day 7 compared with group P (unadjusted *p* < 0.05). At day 15, relative abundances of *Levilactobacillus*, *Lactobacillus*, *Lacticaseibacillus*, and *Bacillus* differed among treatments (unadjusted *p* < 0.05), whereas the remaining dominant genera (*Lentilactobacillus*, *Limosilactobacillus*, and *Weissella*) showed no notable differences.

**Figure 5:**
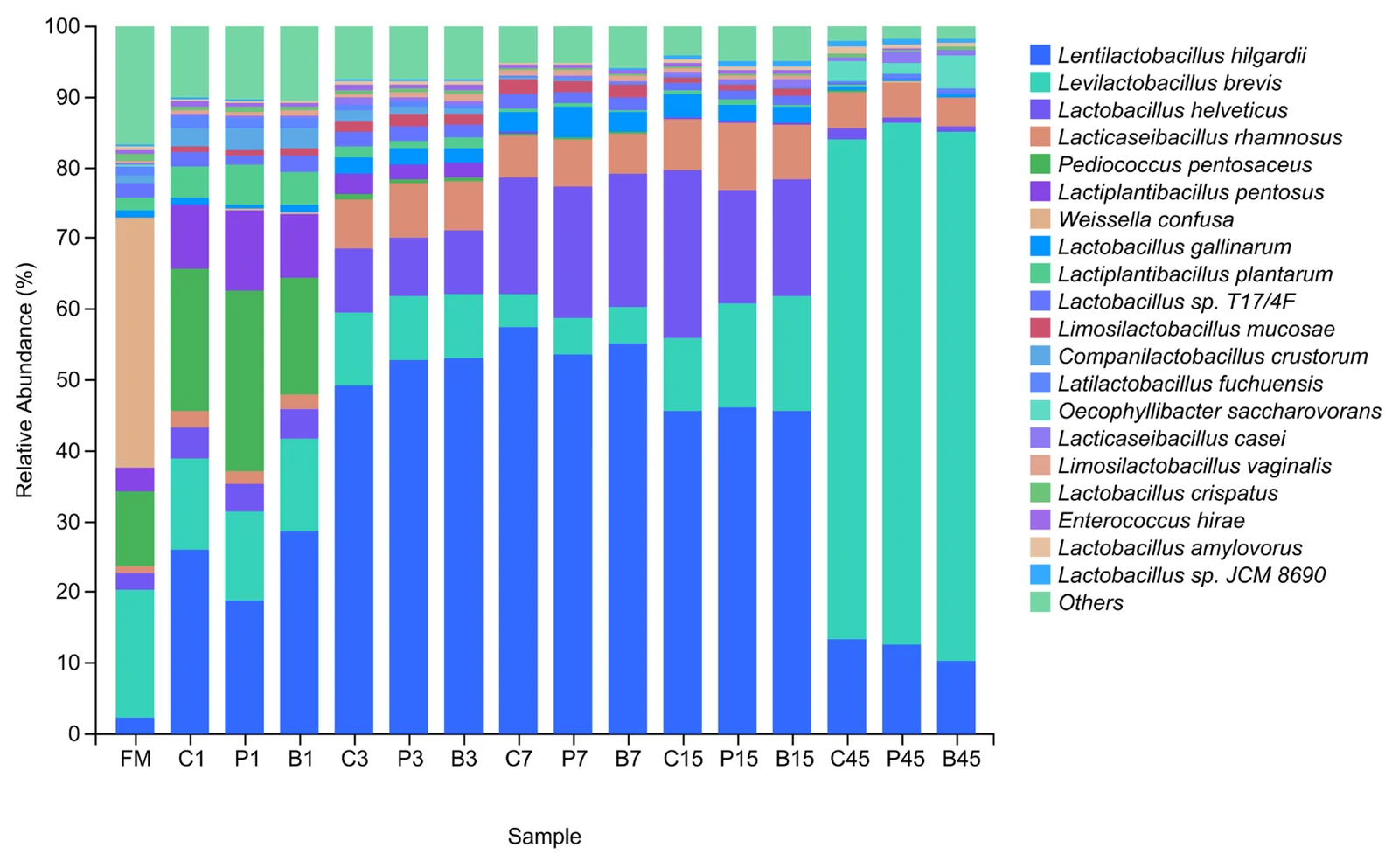
Bacteria at species-level of silage. FM, fresh matter; C, control, with no lactic acid bacteria inoculation; P, silage inoculated with *L. plantarum* LP1; B, silage inoculated with *L. buchneri* 9-2. Samples are labeled accordingly (e.g., C1, P1, B1 for day 1; C3, P3, B3 for day 3; etc.).

Figure 5 shows the species-level bacterial composition in silage. In the fresh materials, *Weissella confusa* (*W. confusa*)*, Levilactobacillus brevis* (*L. brevis)*, and *Pediococcus pentosaceus* (*P. pentosaceus*) were the dominant species, with relative abundances of 35.31%, 18.05%, and 10.41%, respectively. The relative abundance of *L. plantarum* was relatively low (1.90%). Compared with fresh raw materials, the relative abundance of *W. confusa* sharply decreased to below 1% on ensiling day 1. Across all treatment groups, the relative abundances of *Lentilactobacillus hilgardii* (*L. hilgardii*) and *P*. *pentosaceus* showed a marked increase, whereas *L*. *plantarum* maintained a relative abundance consistently exceeding 4.5%. After day 3 of ensiling, *P. pentosaceus* was gradually replaced by *L. hilgardii* and *Lactiplantibacillus pentosus* (*L. pentosus*). After 45 days of ensiling, *L*. *brevis* dominated the bacterial community.

**Figure A2:**
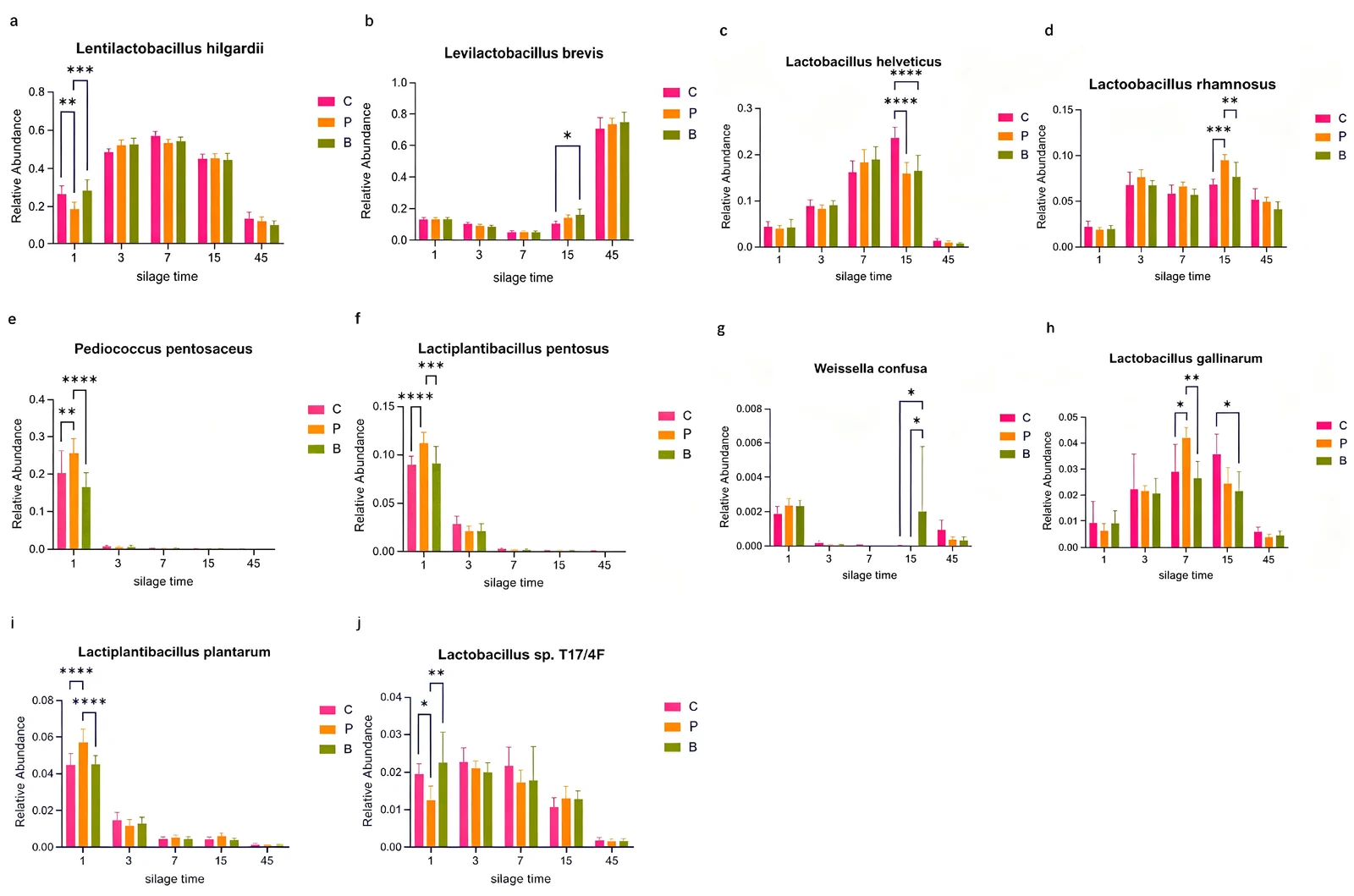
Relative abundance of dominant bacterial species in silage. Panels (**a**–**j**) show the relative abundance (mean ± SD, n = 4) of ten bacterial species: (**a**) *Lentilactobacillus hilgardii*, (**b**) *Levilactobacillus brevis*, (**c**) *Lactobacillus helveticus*, (**d**) *Lacticaseibacillus rhamnosus*, (**e**) *Pediococcus pentosaceus*, (**f**) *Lactiplantibacillus pentosus*, (**g**) *Weissella confusa*, (**h**) *Lactobacillus gallinarum*, (**i**) *Lactiplantibacillus plantarum*, (**j**) *Lactobacillus* sp. *T17/4F*. C, control, with no lactic acid bacteria inoculation; P, silage inoculated with *L. plantarum* LP1; B, silage inoculated with *L. buchneri* 9-2. Samples are labeled accordingly (e.g., C1, P1, B1 for day 1; C3, P3, B3 for day 3; etc.). Inoculation dose: 5 × 10^6^ CFU/g fresh matter. Data were analyzed by one-way ANOVA followed by Tukey’s HSD test for multiple comparisons. * indicates 0.01 < *p* ≤ 0.05; ** indicates 0.001 < *p* ≤ 0.01; *** indicates *p* ≤ 0.001; **** indicates *p* ≤ 0.0001.

Figure A2 presents the relative abundances of the top 10 bacterial species in these silages. On ensiling day 1, group P exhibited lower relative abundances of *L. hilgardii* and *Lactobacillus* sp. T17/4F compared with groups C and B (unadjusted *p* < 0.05). In contrast, the relative abundances of *P. pentosaceus*, *L. pentosus*, and *L. plantarum* were higher in group P than in the other two treatments (unadjusted *p* < 0.05). In addition, group P had higher relative abundances of *Lactobacillus gallinarum* on day 7 and *Lacticaseibacillus rhamnosus* on day 15 than groups C and B (unadjusted *p* < 0.05). On ensiling day 15, groups P and B had lower relative abundances of *Lactobacillus helveticus* than group C (unadjusted *p* < 0.05). However, group B exhibited higher relative abundances of *L*. *brevis* and *W*. *confusa* than group C (unadjusted *p* < 0.05).

### 3.6. Results of Bacterial Differences

**Figure 6:**
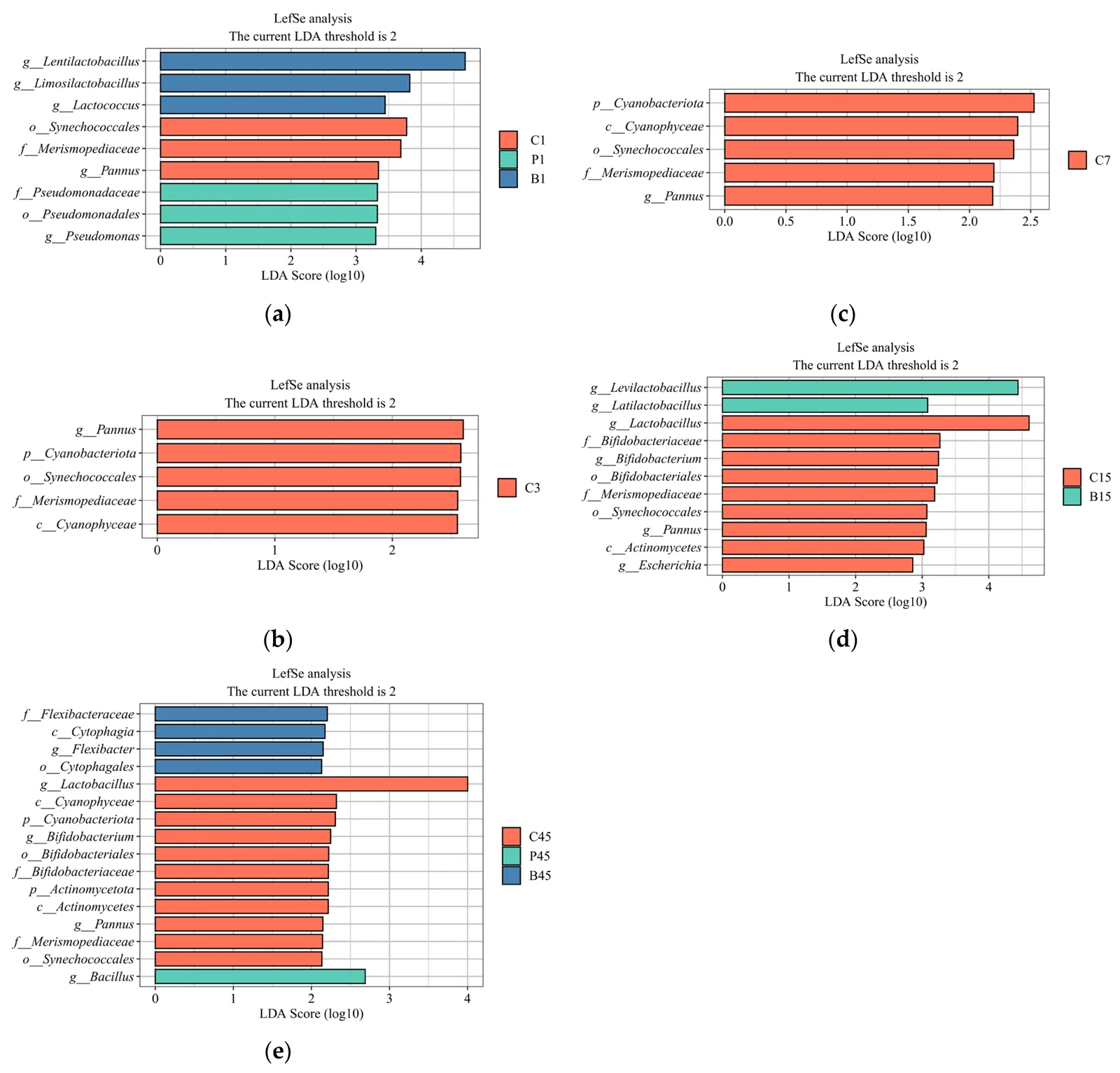
LEfSe results of silage. Figures (**a**–**e**) show the results of LEfSe analysis of bacterial community composition in silage samples after 1, 3, 7, 15, and 45 days of fermentation, respectively. C, control, with no lactic acid bacteria inoculation; P, silage inoculated with *L. plantarum* LP1; B, silage inoculated with *L. buchneri* 9-2. Samples are labeled accordingly (e.g., C1, P1, B1 for day 1; C3, P3, B3 for day 3; etc.). LEfSe analysis was performed independently at each time point using the Python LEfSe package, with Kruskal–Wallis test, one-against-all median-abundance strategy, pairwise Wilcoxon tests, and LDA score > 2.0 as the threshold for biomarker identification. No additional FDR correction was applied.

No additional FDR correction was applied across taxa; therefore, the LEfSe results should be considered exploratory. To investigate differences in taxonomic abundance among groups, LEfSe analysis was performed (Figure 6). After 1 day of silage fermentation, differences in bacterial genera were observed among groups C, P, and B. In silage samples from group B, *Lentilactobacillus*, *Limosilactobacillus*, and *Lactococcus* were identified as potential differentially abundant genera. In contrast, *Pseudomonas* was the potential differentially abundant genus detected in silage samples from group P. On day 15 of silage fermentation, *Levilactobacillus* and *Latilactobacillus* were detected as potential discriminant taxa in silage samples from group B. For the control group, *Lactobacillus* and *Bifidobacterium* were identified as potential differentially abundant genera. After 45 days of silage fermentation, *Lactobacillus* and *Bifidobacterium* were potential differentially abundant taxa in group C; *Bacillus* in group P; and *Flexibacter* in group B.

### 3.7. Prediction of Potential Functions

**Figure 7:**
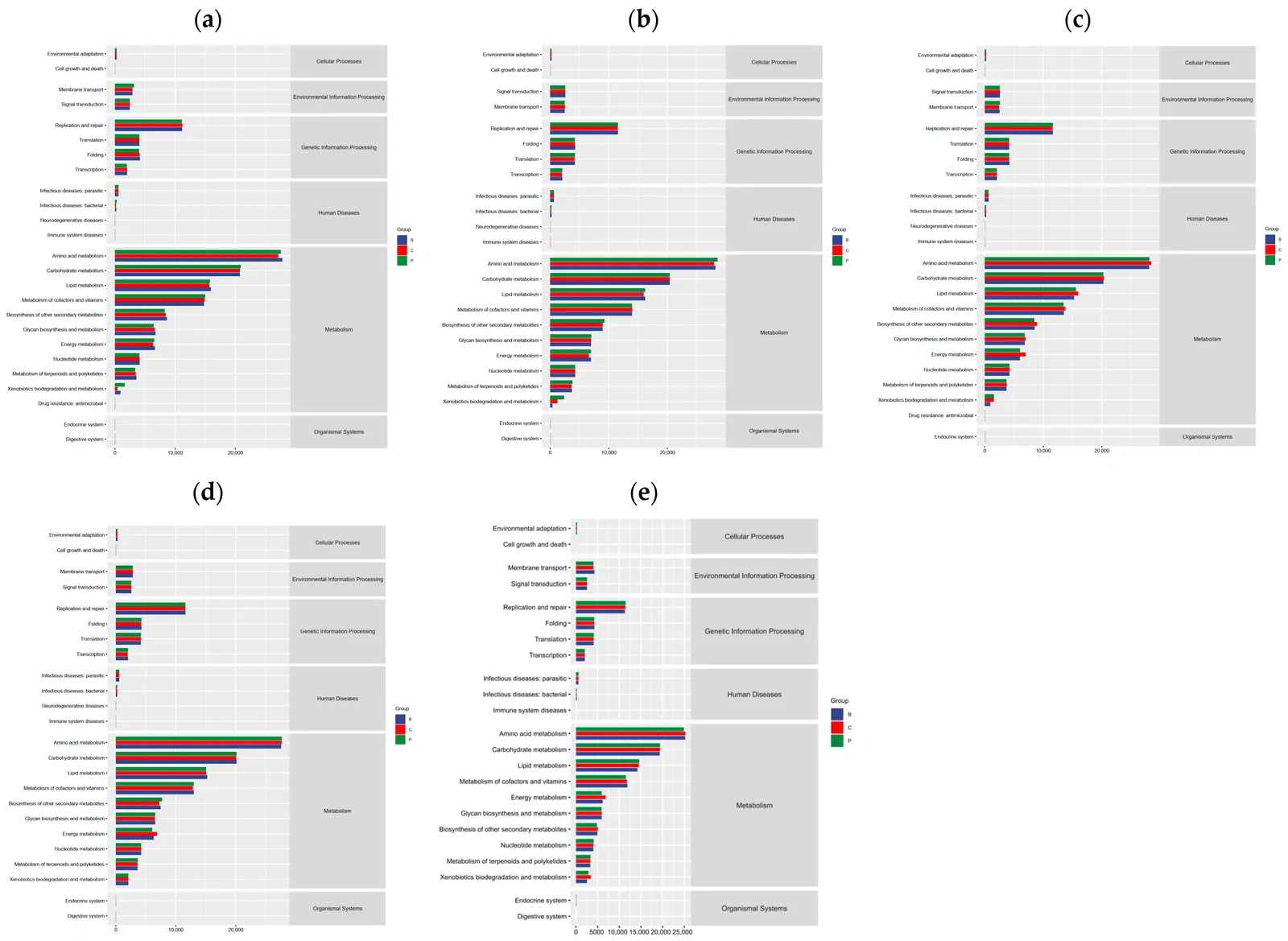
Functional abundance map of KEGG secondary functional predictions for fresh sweet corn processing by-products. Figures (**a**–**e**) show the KEGG secondary functional predictions based on bacterial community composition in silage samples after 1, 3, 7, 15, and 45 days of fermentation, respectively. C, control, with no lactic acid bacteria inoculation; P, silage inoculated with *L. plantarum* LP1; B, silage inoculated with *L. buchneri* 9-2. Samples are labeled accordingly (e.g., C1, P1, B1 for day 1; C3, P3, B3 for day 3; etc.).

No statistical comparisons were performed on the predicted functional profiles; the results are presented descriptively as inferred functional potential. As shown in Figure 7, KEGG secondary functional prediction analysis revealed that metabolism-related pathways were the most abundant categories in the predicted functional profiles of fresh sweet corn by-product silage across all samples. Among all annotated pathways, amino acid metabolism, carbohydrate metabolism, cofactor and vitamin metabolism, and lipid metabolism were the most abundant functional categories. With the progression of ensiling, the predicted relative abundances of amino acid metabolism and xenobiotic biodegradation and metabolism showed a gradual increase. The predicted relative abundance of amino acid metabolism appeared to be higher in groups P and B than in group C on days 1 and 3 of ensiling.

## 4. Discussion

### 4.1. The Effect of Lactic Acid Bacteria on Silage Fermentation Quality

The rapid proliferation of lactic acid bacteria at the initial stage of ensiling, and the bacterial community that subsequently establishes, are critical to the fermentation process and final silage quality [28]. It is generally accepted that the first three days of silage fermentation represent a period of rapid pH reduction, which is crucial for inhibiting harmful microorganisms and minimizing nutrient losses [29]. Research by Ni et al. [30] reported that *Streptococcus*, *Lactococcus*, and *Weissella*, as lactic acid-producing bacteria, initiate lactic acid fermentation at the early stage of ensiling. Previous studies have reported that pH begins to decrease within 12 h after the onset of anaerobic fermentation in whole-plant corn silage [31]. In the present experiment, silage pH in all groups dropped below 4.2 on day 1 (group C: 3.72; group P: 3.66; group B: 3.62) and reached its lowest value (<3.5) by day 45. This rapid acidification is likely attributable to abundant lactic acid bacteria in the fresh sweet corn by-product substrate, predominantly *Weissella* (37.82%), *Lactiplantibacillus* (18.06%), *Pediococcus* (10.86%), and *Lactobacillus* (8.58%). These native microbial communities were associated with rapid fermentation after ensiling. *P. pentosaceus* acts rapidly at the early fermentation stage, producing sufficient lactic acid within a short period to reduce pH [32]. Guo et al. [33] reported that acid-tolerant *Lactobacillus* replaced early colonizers such as *Enterococcus* during the later stages of ensiling, representing a general successional pattern in silage fermentation. On day 1, group P exhibited higher abundances of *P. pentosaceus*, *L. pentosus*, and *L. plantarum*, which coincided with accelerated lactic acid production. *L. gallinarum* (day 7) and *L. rhamnosus* (day 15) were also more abundant in group P, suggesting that acid-tolerant strains may contribute to maintaining the acidic environment. By day 45, *L. brevis* became dominant, which contributed to further pH reduction. Throughout the 45-day ensiling period, the sustained dominance of lactic acid bacteria—involving multiple species such as *P. pentosaceus*, *L. pentosus*, and *L. plantarum* that may have acted synergistically or sequentially—was likely associated with substantial organic acid accumulation and a final pH below 3.5, with group P exhibiting significantly lower pH than that in the other two groups.

*L. plantarum* has been reported to effectively reduce the NH_3_-N ratio in silage via multiple mechanisms, including reducing the rate of protein catabolism and enhancing energy utilization efficiency [34]. During silage fermentation, pathways involved in carbohydrate metabolism, nucleotide metabolism, energy metabolism, amino acid metabolism, and cofactor and vitamin metabolism are closely associated with fermentation characteristics and changes in the chemical composition of silage [35]. Kilstrup et al. [36] reported that most bacterial metabolic reactions involve either nucleotide utilization or metabolite-mediated regulation of nucleotide metabolism. Previous studies have reported that enhanced amino acid metabolism is associated with the moderate degradation of crude protein in silage [37]. The resulting metabolic intermediates further promote organic acid accumulation and accelerate pH decline, thereby exerting a notable inhibitory effect on protein degradation triggered by endogenous proteases and spoilage microorganisms [38]. The PICRUSt2-based functional prediction in this study showed that the predicted relative abundance of amino acid metabolic pathways increased with ensiling time, coinciding with a gradual decrease in NH_3_-N. This suggests that *L. plantarum* might reduce NH_3_-N levels through enhanced amino acid metabolism and inhibited proteolysis; however, confirmatory studies are needed to substantiate this possibility.

In the present experiment, neither PA nor BA was detected in silage prepared from fresh sweet corn processing by-products after 45 days of ensiling. Several studies have reported that inoculation with *L. buchneri* reduces propionic acid concentrations, and similar findings have been observed in whole-plant corn silage after 90 days of fermentation [39]. Some studies have also demonstrated that PA in whole-plant corn silage is only detectable after 60 days of ensiling [31]. Furthermore, pH remained consistently below 4.2 throughout the ensiling period in the present study. This may have been unfavorable for propionic acid- and butyric acid-producing microorganisms, such as *Propionibacterium* and *Clostridium*, which typically require a pH above 4.0–4.5 for growth and metabolism [40]. Alternatively, even if propionic acid was produced in trace amounts, its concentration may have fallen below the detection limit, preventing accurate quantification. Functional prediction revealed that carbohydrate metabolic pathways exhibited the highest relative abundance across all samples. This aligns with the efficient conversion of water-soluble carbohydrates into lactic acid (LA) and acetic acid (AA) during ensiling [14]. This is corroborated by the significant increases in LA and AA concentrations throughout fermentation. Previous studies demonstrated that *L. buchneri* degrades lactic acid under anaerobic acidic conditions [12]. However, species-level sequencing results from the present study revealed that *L. buchneri* was not detected in any group throughout the ensiling period, suggesting that the inoculated strain failed to become a dominant or persistent member of the bacterial community. A possible explanation is that the fresh sweet corn by-product feedstock harbored abundant epiphytic lactic acid bacteria, which may have placed the inoculated *L. buchneri* at a competitive disadvantage and restricted its colonization and survival within the silage ecosystem. In contrast, *L. plantarum* inoculated into group P rapidly colonized and dominated the fermentation, which coincided with a sharp decline in pH. Before day 7 of fermentation, acetic acid concentration in group B was higher than that in group P, which is consistent with the metabolic characteristics of heterofermentative lactic acid bacteria that utilize both hexoses and pentoses to produce lactic acid and acetic acid simultaneously. However, this trend reversed by day 15, with acetic acid concentrations in group P remaining consistently higher thereafter (Figure 1d). Two potential explanations may account for this observation. First, indigenous heterofermentative lactic acid bacteria may remain metabolically active even under the low-pH conditions established by *L. plantarum* and may continue to produce acetic acid. Second, although *L. plantarum* is facultatively heterofermentative, it can also produce small amounts of acetic acid on its own under specific substrate- or oxygen-limited conditions. Consequently, during the late fermentation stage, both lactic acid and acetic acid concentrations in group P were significantly higher than those in group B.

### 4.2. The Effects of Lactic Acid Bacteria on Chemical Composition and In Vitro Digestibility

In the present study, no significant differences were observed in DM or CP concentrations among groups after 45 days of ensiling, suggesting that, under the tested conditions, the inoculation of exogenous lactic acid bacteria did not substantially alter these two nutritional parameters. Changes in protein concentration during ensiling are primarily governed by the proteolytic activity of endogenous plant proteases and that of spoilage microorganisms, both of which exhibit optimal activity at pH 5–7 [41]. *L. plantarum* has been reported to promote rapid acidification, reducing the pH to below 4.2, which may suppress the activity of these enzymes and microorganisms, thereby reducing protein hydrolysis and amino acid deamination without altering the total crude protein concentration [42]. This result is consistent with the observed decrease in NH_3_-N concentration. This view is also corroborated by studies on *Silphium perfoliatum* silage [43]. However, as CP is expressed on a dry matter basis, these results do not directly prove reduced absolute protein losses. No significant changes in NDF or ADF concentrations were observed among groups, but ADL concentration was significantly lower in groups P and B than in the control group. This apparent decrease does not necessarily imply actual lignin degradation. Lignin is generally resistant to microbial degradation under anaerobic silage conditions [44]. The observed reduction in ADL may be partly attributed to greater organic acid accumulation in the inoculated silage, which could partially cleave lignin–carbohydrate ester bonds, increasing acid-soluble extraction and reducing residual ADL [45]. The unchanged NDF and ADF support this view, as true fiber degradation would affect multiple fractions, not ADL alone. However, because dry matter losses and water-soluble carbohydrate dynamics were not measured in the present study, the exact mechanism underlying the ADL reduction cannot be fully confirmed, and this interpretation should be considered tentative.

Although no significant differences were observed in fiber components (NDF and ADF) among groups, the IVOMD, IVCPD, and IVNDFD values in group P remained significantly higher than those in groups C and B. Studies have shown that the *in vitro* digestibility of legume silage is significantly improved by the addition of *L. plantarum* [46]. The acidic microenvironment formed following inoculation with *L. plantarum* may facilitate the retention of greater amounts of digestible nutrients. Contreras-Govea et al. found that compared with the control group, inoculation with *L. plantarum* (Lp, strain MTD/1) retained higher true protein concentrations during ensiling; the elevated true protein concentration promoted the proliferation of ruminal microorganisms, thereby improving *in vitro* digestibility [47]. It should be noted that all *in vitro* incubations in the present experiment were performed in a single run using pooled rumen fluid from four fistulated sheep. This approach is commonly used for *in vitro* digestibility assays to minimize variation among donor animals and to ensure consistent inoculum conditions across all groups. However, the statistical comparisons only reflect differences among silages under this single inoculum batch; future studies using multiple independent rumen inoculum sources are required to verify these findings.

### 4.3. The Effect of Lactic Acid Bacteria on Oxygen Stability

In the present study, all groups maintained aerobic stability above 72 h, with no significant intergroup differences (*p* = 0.065), although a numerical trend of C < P < B was observed. This may be attributed to the small sample size (*n* = 4) and the high baseline stability of the control group (76 h), which limited the observed treatment effects. As mentioned above (Section 4.1), *L. buchneri* was not detected throughout the ensiling period, suggesting that it did not become an established member of the community. This phenomenon may partly explain why inoculation with *L. buchneri* did not significantly improve aerobic stability in the present experiment. Alternatively, the undetectability may also reflect the inherent limitations of 16S rRNA sequencing in resolving closely related species at the species level, leaving open the possibility that *L. buchneri* persisted at extremely low abundance. If the inoculated lactic acid bacteria fail to rapidly establish ecological dominance, yeasts may quickly revive after silo opening, consume lactic acid, and initiate secondary fermentation [48], thereby reducing aerobic stability. Additionally, the 45-day ensiling period may have been too short for *L. buchneri* to fully express its heterofermentative activity, and strain-specific traits of *L. buchneri* 9-2 may have also constrained its colonization and acetic acid production in this substrate. AA inhibits yeasts and molds, whereas LA is preferentially utilized by these microorganisms under aerobic conditions [49,50]. The substantial accumulation of LA in group P, upon air exposure, may have provided readily utilizable energy sources for spoilage microorganisms, thereby partially offsetting the inhibitory effect of AA. In contrast, the lower concentrations of both organic acids in group B may have resulted in relatively weaker microbial activity after bag opening, which might explain its numerically higher aerobic stability. However, given the lack of statistically significant differences among groups and the absence of quantitative data on yeasts and spoilage bacteria in the present study, the above interpretation remains speculative.

### 4.4. The Effects of Lactic Acid Bacteria Preparations on the Bacterial Community

The Shannon index is one of the most commonly used measures for evaluating alpha diversity. Generally, high-quality silage tends to have lower microbial alpha diversity [51]. The results of the present experiment showed that the Shannon index in all groups reached its lowest level after 45 days of fermentation. PCoA showed that samples from different ensiling days formed distinct clusters. PERMANOVA further confirmed that ensiling day was the dominant factor shaping bacterial community composition, whereas the effect of inoculation was limited. Therefore, natural microbial succession, rather than inoculation, appears to be the main factor associated with community dynamics during ensiling, with temporal factors—such as progressive pH decline, substrate depletion, and altered oxygen availability—likely to shape the microbial community.

The abundant indigenous microorganisms in the initial ensiling system may suppress the proliferation of exogenous lactic acid bacteria [52]. In addition to competitive exclusion by indigenous microorganisms (see Section 4.1), environmental factors during anaerobic fermentation—such as temperature and organic acid accumulation—may also influence the survival of the inoculated strain [13], although these parameters were not directly evaluated here. Studies have shown that when whole-plant corn silage is ensiled for 45–60 days at 30 °C and inoculated with *L. plantarum* or *L. buchneri*, the bacterial diversity of the silage differs from that of the control group; however, no such difference is observed at 20 °C [31]. In the present experiment, the sealed bags were stored at ambient room temperature (20.5 ± 1.5 °C), which falls within the range where inoculation effects on bacterial diversity were not pronounced in previous reports.

The abundance and composition of microorganisms vary with silage raw material type and the ensiling process [53]. At the genus level, microbial composition underwent substantial changes before and after ensiling, and bacterial diversity gradually decreased as ensiling time progressed. Studies have demonstrated that inoculation of whole-plant corn silage with *L. plantarum* can significantly reduce bacterial diversity [54]. In the present experiment, *Weissella* exhibited a high relative abundance (37.82%) before ensiling, but its abundance dropped sharply after day 1, suggesting poor acid tolerance. Yang et al. [28] reported that the activity of *Weissella* is inhibited under low-pH conditions, a finding that supports the above inference. Dong et al. [27] reported that, at the genus level, the dominant genera in whole-plant corn silage after 42 days of ensiling included *Lactobacillus*, *Weissella*, and *Klebsiella*, with their combined relative abundance exceeding 75%. This discrepancy may be attributed to factors such as silage type, variety, chemical composition, growth environment, and regional differences [55]. In addition to *Lactobacillus*, *Weissella*, *Pediococcus*, *Enterococcus*, and *Leuconostoc* accounted for a significant proportion of the microbial community in corn silage [56]. In the present study, *Pediococcus* exhibited the highest relative abundance on day 1, reaching 26.55%. *Pediococcus* is a homofermentative genus capable of rapid lactic acid production, and its high abundance coincided with the early pH decline in this study. After 45 days of ensiling, *Levilactobacillus* became the dominant genus in all treatment groups. As a heterofermentative genus, *Levilactobacillus* may have contributed to acetic acid accumulation through its predominance in the later stage and may have helped maintain fermentation stability during extended storage.

At the species level, *W. confusa* decreased sharply on day 1 of ensiling, while *L. hilgardii* and *P. pentosaceus* became dominant. However, after day 3, *P. pentosaceus* was gradually replaced by *L. hilgardii* and *L. pentosus*. Inoculation with *L. plantarum* increased the relative abundances of *L. plantarum*, *P. pentosaceus*, and *Lactiplantibacillus pentosus* on day 1 of ensiling. *L. plantarum* exhibited a low abundance (1.90%) in the fresh raw material, but its relative abundance remained above 4.5% throughout the ensiling period in the inoculated group, which is consistent with its association with sustained acid production and pH maintenance [37]. *L. plantarum* exhibited a low abundance (1.90%) in the fresh raw material, but its relative abundance remained above 4.5% throughout the ensiling period in the inoculated group, which is consistent with its association with sustained acid production and pH maintenance. *L. hilgardii* maintained a relatively high abundance across all groups throughout the ensiling period, with an overall trend of initially increasing and then decreasing. As a heterofermentative strain, it is associated with the production of both lactic acid and acetic acid throughout the ensiling process [50]. Previous studies have reported that inoculation with *L. buchneri* or *L. plantarum* increased the relative abundance of *L. brevis* in silage from 3 to 30 days compared with the control group [31,57]. In the present experiment, the relative abundance of *L. brevis* in groups P and B peaked at 45 days. Although this bacterium is heterofermentative, it has a broad carbohydrate utilization spectrum and becomes dominant in late fermentation [58]. Thus, it may be associated with system stability and acetic acid accumulation in the late fermentation stage.

Several limitations of the present study should be noted. First, only one strain per species was tested, limiting generalizability. Second, the sample size was moderate (*n* = 4 per group). Third, species-level taxonomic assignments based on 16S rRNA sequencing are inherently constrained by database limitations and a lack of genomic validation. Fourth, functional predictions were not experimentally verified. Finally, no animal feeding trial was performed, so the *in vitro* findings await in vivo confirmation. Nonetheless, the present study offers a controlled evaluation of the two inoculants and provides a foundation for further research.

## 5. Conclusions

In conclusion, *L. plantarum* improved the fermentation quality and *in vitro* digestibility of fresh sweet corn processing by-product silage more effectively than *L. buchneri*. Although the improvement in aerobic stability did not reach statistical significance, both inoculants showed a numerical increase relative to the control. The fresh sweet corn by-product feedstock was initially dominated by *Weissella*, *Levilactobacillus*, and *Lactobacillus*. On day 1 of ensiling, *Pediococcus* and *Lentilactobacillus* rapidly became dominant, coinciding with the drop in pH during acidification. The dominance of *Pediococcus* disappeared by day 3; shifts in the abundance of *L. plantarum* and *L. hilgardii* were observed alongside sustained acid production throughout fermentation, and *L. brevis* became dominant in the later stage. Inoculation with *L. plantarum* also altered the relative abundance of certain bacterial taxa at specific time points. Overall, under the laboratory-scale conditions tested (500 g per bag), *L. plantarum* exhibited superior performance in improving silage fermentation quality and *in vitro* digestibility compared with *L. buchneri*. These findings provide a basis for larger-scale ensiling trials and in vivo animal studies to further validate the benefits observed under laboratory conditions.

## Figures and Tables

**Table 1:** Characteristics, buffering capacity and gross energy of fresh corn by-products and wheat bran.

Item ^1^	Fresh Corn Processing By-Products	Wheat Bran
DM (%FW)	22.98 ± 0.076	86.25 ± 0.356
OM (%DM)	98.25 ± 0.057	94.40 ± 0.300
CP (%DM)	10.32 ± 0.163	15.79 ± 0.540
NDF (%DM)	70.89 ± 0.990	52.06 ± 0.596
ADF (%DM)	24.97 ± 0.425	16.75 ± 0.537
ADL (%DM)	6.70 ± 0.267	4.69 ± 0.285
WSC (%DM)	7.03 ± 0.177	7.88 ± 0.305
BC (mEq/kg DM)	194.28 ± 1.937	110.03 ± 0.506
GE (MJ/kg DM)	18.96 ± 0.820	18.44 ± 0.472

^1^ All values are expressed as means ± SEM (*n* = 4); DM, dry matter; FW, fresh weight; OM, organic matter; CP, crude protein; NDF, neutral detergent fiber; ADF, acid detergent fiber; ADL, acid detergent lignin; WSC, water-soluble carbohydrate; BC, buffering capacity; GE, gross energy.

**Table 2:** Chemical composition of fresh corn silage ensiled for 45 d.

Item ^1^	Additives ^2^			SEM ^3^	*p*-Value
	C	P	B		
DM (%FW)	31.97	31.76	31.39	0.421	0.419
OM (%DM)	94.80 ^a^	94.74 ^a^	95.56 ^b^	0.084	<0.001
CP (%DM)	13.22	14.01	13.64	0.394	0.485
NDF (%DM)	53.61	55.22	53.09	0.877	0.089
ADF (%DM)	19.50	20.43	19.53	0.491	0.154
ADL (%DM)	6.17 ^b^	4.52 ^a^	4.63 ^a^	0.310	0.001

^a,b^ Different lowercase letters in the same row indicate differences (*p* < 0.05). ^1^ DM, dry matter; FW, fresh weight; OM, organic matter; CP, crude protein; NDF, neutral detergent fiber; ADF, acid detergent fiber; ADL, acid detergent lignin. ^2^ C, control, with no lactic acid bacteria inoculation; P, inoculated with *L. plantarum* LP1 at 5 × 10^6^ CFU/g fresh matter; B, inoculated with *L. buchneri* 9-2 at 5 × 10^6^ CFU/g fresh matter. ^3^ SEM, standard error of the mean. Data were analyzed by one-way ANOVA followed by Tukey’s HSD test for multiple comparisons (*n* = 4).

**Table 3:** *In vitro* digestibility of silage ensiled for 45 d.

Item ^1^	Additives ^2^			SEM ^3^	*p*-Value
	C	P	B		
IVDMD (%)	64.81 ^ab^	64.28 ^a^	65.31 ^b^	0.279	0.015
IVOMD (%)	69.53 ^a^	70.45 ^b^	70.09 ^a^	0.288	0.032
IVCPD (%)	57.65 ^a^	58.50 ^b^	58.09 ^a^	0.335	0.042
IVNDFD (%)	68.33 ^a^	69.36 ^b^	68.33 ^a^	0.274	0.014
IVGED (%)	49.08	50.64	48.58	0.847	0.089

^a,b^ Different lowercase letters in the same row indicate differences (*p* < 0.05). ^1^ IVDMD, *in vitro* dry matter digestibility; IVOMD, *in vitro* organic matter digestibility; IVCPD, *in vitro* crude protein digestibility; IVNDFD, *in vitro* neutral detergent fiber digestibility; IVGED, *in vitro* gross energy digestibility. ^2^ C, control, with no lactic acid bacteria inoculation; P, inoculated with *L. plantarum* LP1 at 5 × 10^6^ CFU/g fresh matter; B, inoculated with *L. buchneri* 9-2 at 5 × 10^6^ CFU/g fresh matter. ^3^ SEM, standard error of the mean. Data were analyzed by one-way ANOVA followed by Tukey’s HSD test for multiple comparisons (*n* = 4).

## Data Availability

The original contributions presented in this study are included in the article and Appendix A. For further inquiries, please contact the corresponding author upon reasonable request.

## Appendix A

**Table A1:** Daily ration formula and chemical composition of feed.

Item	Ratio/Content (%DM)
Dietary Formula	
Corn silage (%)	55
Chinese wildrye (%)	15
Flaxseed meal (%)	5
Corn germ meal (%)	5
Concentrate feed (%) §	20
Chemical Composition	
DM (%FW)	88.33
CP (%DM)	11.67
NDF (%DM)	38.42
ADF (%DM)	17.88
EE (%DM)	2.77
Ca (%DM)	0.91
Total phosphorus (%DM)	0.35

§ Concentrate components: corn 55.0%, soybean meal 10.0%, canola meal 11.0%, cotton meal 7.0%, corn protein meal 8.0%, soda 1.0%, stone powder 2.2%, magnesium oxide 0.8%, ammonium chloride 1.0%, calcium bicarbonate 1.0%, sodium chloride 2.0%, compound premix 1.0%. DM, dry matter; FW, fresh weight; CP, crude protein; NDF, neutral detergent fiber; ADF, acid detergent fiber; EE, ether extract.

**Table A2:** Read statistics at each bioinformatic processing step for 16S rRNA gene sequencing of all samples.

Sample ID	Raw Reads (Input)	Filtered	Retained Reads (Denoised)	Non-Chimeric	Non-Singleton
CK1_1	60572	41308	39583	36696	17648
CK1_2	62390	42472	40749	37823	17522
CK1_3	65407	44847	42850	40243	18623
CK1_4	60839	41446	39719	37632	16848
P1_1	63466	43392	41708	40394	16812
P1_2	59864	41025	39417	37955	14682
P1_3	62965	42917	41194	39039	16638
P1_4	62349	42707	40931	39180	15725
B1_1	62435	42552	40836	38600	17159
B1_2	59748	40831	39138	36555	17143
B1_3	64656	44105	42314	39840	17626
B1_4	65262	44434	42629	39948	18253
CK3_1	61290	42043	40370	36847	26097
CK3_2	62397	42602	40625	37644	27454
CK3_3	62429	42551	40667	38047	25280
CK3_4	62014	42630	40656	38079	25682
P3_1	65616	44903	42869	40201	28488
P3_2	63739	43625	41365	39544	29298
P3_3	65762	45041	42839	40714	27819
P3_4	65525	44583	42568	38943	26609
B3_1	65472	44543	42635	38472	28213
B3_2	60657	41538	39689	36992	26580
B3_3	63722	43626	41423	39233	24219
B3_4	59898	40979	39128	35854	27290
CK7_1	61012	41843	40280	35814	31046
CK7_2	63000	43056	41642	35681	31522
CK7_3	63031	43055	41422	36988	32235
CK7_4	62200	42580	41042	36389	31898
P7_1	61251	41766	40108	36999	31678
P7_2	60684	41376	39831	35833	31255
P7_3	59234	40211	38648	36508	31745
P7_4	64094	43756	41947	38022	33477
B7_1	61241	41999	40296	36836	32004
B7_2	65639	45106	43192	39272	34062
B7_3	61584	42226	40673	37205	32389
B7_4	65260	44696	43289	38042	34147
CK15_1	64766	44121	43082	31645	28966
CK15_2	59773	40748	39630	31055	28280
CK15_3	63021	43038	41917	35115	32336
CK15_4	62245	42341	41184	33644	30635
P15_1	60527	41171	39906	32854	29914
P15_2	62845	42892	41421	35936	32268
P15_3	60566	41538	40358	32924	30035
P15_4	65452	44882	43683	35754	32695
B15_1	59537	40721	39537	31330	28217
B15_2	59705	40503	39199	33216	29870
B15_3	62937	43015	41854	32183	29478
B15_4	63622	43363	42339	32566	28687
CK45_1	59447	40414	40048	36630	35255
CK45_2	60063	40811	40539	38285	37106
CK45_3	61257	41542	41195	35961	34213
CK45_4	61582	41948	41674	39860	38631
P45_1	59514	40517	40244	39055	37960
P45_2	67429	64222	63876	60554	59040
P45_3	64752	44252	43963	41781	40627
P45_4	61502	41975	41670	38921	37627
B45_1	64389	43839	43539	37969	36659
B45_2	63901	43501	43193	38590	37156
B45_3	60514	41450	41304	39080	38070
B45_4	61793	42136	41982	40163	39290
FW_1	60271	41347	40134	35764	16074
FW_2	62296	42883	41579	36973	16073
FW_3	60263	41314	40082	35481	16467
FW_4	61525	42313	41075	36429	16770

Raw reads (Input) represent the original sequencing output from the Illumina platform. Filtered reads were obtained after primer trimming and quality filtering. Denoised reads were the retained reads. Non-chimeric reads and non-singleton reads were obtained after chimera removal and singleton filtering, respectively. CK, control, with no lactic acid bacteria inoculation; P, silage inoculated with *L. plantarum* LP1; B, silage inoculated with *L. buchneri* 9-2. Samples are labeled accordingly (e.g., C1, P1, B1 for day 1; C3, P3, B3 for day 3; etc.).
